# *Cryptosporidium* spp. Infections in Combination with Other Enteric Pathogens in the Global Calf Population

**DOI:** 10.3390/ani11061786

**Published:** 2021-06-15

**Authors:** Beate Conrady, Michael Brunauer, Franz-Ferdinand Roch

**Affiliations:** 1 Institute of Food Safety, Food Technology and Veterinary Public Health, University of Veterinary Medicine, 1210 Vienna, Austria; m.brunauer@gmx.net (M.B.); Franz-Ferdinand.Roch@vetmeduni.ac.at (F.-F.R.); 2 Department of Veterinary and Animal Sciences, Faculty of Health and Medical Sciences, University of Copenhagen, 1870 Frederiksberg, Denmark; 3 Complexity Science Hub Vienna, 1080 Vienna, Austria

**Keywords:** bovine rotavirus, bovine coronavirus, concurrent-infection, *Cryptosporidium* spp., *Escherichia coli* F5 (K99), epidemiology, mixed-infection, neonatal calf diarrhoea, systematic review

## Abstract

**Simple Summary:**

The factors “diagnostic”, “health status of the sampled animals”, and “geographical region” explained the majority of the variance of *Cryptosporidium* spp. (Crypto) prevalence in the global calf population across the literature. The chance of detecting bovine rotavirus (BRV), bovine coronavirus (BCoV), and enterotoxigenic *Escherichia coli* F5 (K99) (ETEC) in calves with diarrhoea was lower in the presence of Crypto compared to calves without Crypto. This may indicate an inhibition effect between BRV, BCoV, ETEC, and Crypto.

**Abstract:**

The most common worldwide diarrhoea-causing agents in neonatal calves are *Cryptosporidium* spp. (Crypto), bovine rotavirus (BRV), bovine coronavirus (BCoV), and enterotoxigenic *Escherichia coli* F5 (K99) (ETEC). Crypto is a zoonotic pathogen of diarrhoea in humans, particularly for children and immunocompromised adults. Four weighted-stratified random-effect meta-analyses including meta-regression analyses were performed to calculate the worldwide mean prevalence of Crypto and associated concurrent infections with BRV, BCoV and ETEC, as well as their potential influencing factors. The meta-analysis incorporated 28 studies (56 substudies) in 17 countries that determined the presence or absence of concurrent infections with Crypto in the global calf population. Approximately half of all considered studies presented here were conducted in Europe independently of the type of infections with Crypto. Within Europe, the highest estimated mean Crypto-BRV prevalence was identified in Ireland (16.7%), the highest estimated mean Crypto-BCoV prevalence was detected in the United Kingdom (4.3%), and the highest estimated mean Crypto-ETEC prevalence across the literature was determined in Turkey (4.7%). The chance of detecting BRV, BCoV, and ETEC in calves with diarrhoea was 0.8 (confidence interval (CI): 0.6–1.0), 0.7 (CI: 0.5–1.0) and 0.6 (CI: 0.4–0.9) lower in the presence of Crypto compared to calves without Crypto. This may indicate an inhibitory effect between BRV, BCoV, ETEC, and Crypto in calves. The variance in the published prevalence across the literature can mainly be explained by the “diagnostic” factor (R^2^ min–max: 0.0–40.3%), followed by the “health status of the sampled animals” (R^2^ min–max: 1.4–27.3%) and “geographical region” (R^2^ min–max: 5.9–23.6%).

## 1. Introduction

The most common worldwide diarrhoea-causing agents in neonatal calves are *Cryptosporidium* spp. (Crypto), bovine rotavirus (BRV), bovine coronavirus (BCoV), and enterotoxigenic *Escherichia coli* F5 (K99) (ETEC) [1,2,3,4]. These enteric pathogens occur frequently as concurrent infections in calves, whereby calves are most susceptible to infections with Crypto in the first three weeks of age in sampled animals [5]. Crypto is a zoonotic pathogen [6] which cause diarrhoea in children younger than 5 years and immunocompromised adults for which no vaccines are available yet [6]. Investigating the geographical distribution, frequency, and influencing factors of (zoonotic) pathogens [7] in production animals is of particular importance to (veterinary) public health authorities, as it can increase the visibility of the distribution of causative agents in the global animal population [8]. 

A published literature study by Brunauer and colleagues [9] analysed the prevalence of mixed infections in calves with bovine rotavirus (BRV), i.e., BRV in combination with BCoV, ETEC, and Crypto. The authors did not investigate the prevalence of Crypto and associated infections with Crypto. Thus, the collected data regarding prevalence, and the statistical approach currently published by Brunauer et al. was used to (i) analyse the geographical distribution of Crypto and associated concurrent infections with Crypto (i.e., Crypto-BRV, Crypto-BCoV, Crypto-ETEC) across the literature, (ii) to determine potential influencing factors (e.g., geographical region, herd type, health status of sampled animals, sample type, diagnostic methods, study type, age of sampled animals) on the Crypto prevalence, and (iii) to calculate the estimated mean prevalence of Crypto from published studies stratified by potential influencing factors in the global calf population by performing weighted random-effect meta-analyses and uni- and multivariate meta-regression analyses. The benefits of a meta-analysis are that such investigations are more powerful and less biased than common statistical approaches of single studies. Thus, a meta-analysis provides an accurate overview of influencing factors on reported prevalence across the literature [8,10,11]. As far as the authors are aware, this is the first meta-analysis to be carried out for mixed infections with Crypto for the worldwide calf population. 

## 2. Materials and Methods

The utilisation of the same dataset currently published by Brunauer and colleagues [9] for the study presented here was possible because the authors used very broad keywords such as neonatal calf diarrhoea OR calf diarrhoea OR diarrheic calves OR diarrhoeic calves OR pre-weaned AND prevalence AND mixed infection OR concurrent infection OR co-infection. The authors identified a large number of studies (*n* = 1216). We used these identified studies and excluded studies based on the procedures shown in Figure S1 and according to PRISMA guidelines (Preferred Reporting Items for Systematic Reviews and Meta-analysis). In contrast to the study by Brunauer and colleagues [9] who focused on mixed infection with BRV, the study presented here focused on mixed infections with Crypto (i.e., Crypto in combination with BRV, BCoV, and ETEC). This includes a complete epidemiological picture of mixed-infection with Crypto. As the literature research focuses on mixed infections with Crypto, studies that published the Crypto prevalence (without considering mixed infections) are not included. Since it is not possible to create a comparable basis of Crypto single infections due to different combinations of tested pathogens, the cumulative prevalence was used for Crypto (i.e., positive Crypto detection, regardless of the presence of another pathogen). 

Besides information about the occurrence of the enteropathogens, data about potential influencing factors (i.e., geographical region, herd type, health status of sampled animals (=calves with or without diarrhoea), sample type (=faecal, autopsy or both), diagnostic methods, study type (=covered case-control-studies; case-studies and other studies); period (=defined as period of begin and end of sampling), age of sampled animals; detail definitions of the factors are provided in Tables S1–S4) were also collected from the studies identified across the literature (Figure S1). The collected prevalence data and data on associated potential influencing factors were analysed in a weighted meta-analysis in order to calculate the estimated mean prevalence across the studies stratified by potential influencing factors. The stratification of the prevalence by potential influencing factors was necessary to identify the possible sources of heterogeneity in the published prevalence.

To determine the significant level of potential influencing factors and their explainable proportion on the variability (R^2^) of the Crypto, Crypto-BRV, Crypto-BCoV, and Crypto-ETEC prevalence across the studies, uni- and multivariate regression analyses were performed. In this context, only significant factors with the lowest Akaike information criteria, corrected for small sample size (AICc) from the univariate meta-regression analysis were included in the multivariate regression analysis.

Additionally, we assessed the expected prevalence of mixed infection with Crypto under assumption of independency of both pathogens (see Formulas (1)–(3)) and calculated the chance (odds ratio (OR)) of detecting BRV, BCoV and ETEC in calves with diarrhoea in the presence of Crypto compared to calves without Crypto.

*P*(Crypto ∩ BRV) = *P*(Crypto) × *P*(BRV) (1)

*P*(Crypto ∩ BCoV) = *P*(Crypto) × *P*(BCoV) (2)

*P*(Crypto ∩ ETEC) = *P*(Crypto) × *P*(ETEC)) (3)

A detailed description of the meta-analysis can be found in the recently published study by Brunauer and colleagues [9] and the calculation of publication bias and identification of outliers across the studies (i.e., case influence diagnostic, funnel plots of the meta-analysis and the forest plots stratified by each included study) can be found in Figures S1–S9. The maps were created with the QGIS Version 3.16. The figures and statistical analyses [12,13] were implemented using the R statistical computing environment (Version 3.4.1 R Foundation for Statistical Computing, Vienna, Austria).

## 3. Results

**Figure 1:**
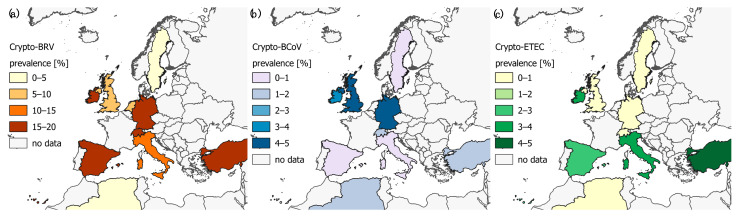
Geographical distribution of mixed-infection of (**a**) Crypto-BRV, (**b**) Crypto-BCoV, and (**c**) Crypto-ETEC. N.B. Other countries were not shown here due to low number of studies but the mean prevalence for all global geographical regions can be found in Tables S1–S4. Table S5 includes the estimated mean prevalence (%) of the pathogens in the countries, shown in Figure 1.

Our study incorporated 28 studies in total (56 substudies i.e., one study can be divided into substudies if e.g., the studies covered prevalence for different herd types) from 1216 identified studies in 17 countries (see Figure S1). The highest mean prevalence for Crypto-BRV, Crypto-ETEC, and Crypto-BCoV was identified in West Asia (Crypto-BRV: 16.6%; CI: 8.0–27.2, Crypto-ETEC: 4.7%; CI: 0.7–11.0) and North America (Crypto-BCoV: 6.7%; CI = 2.4–12.5), respectively (see Tables S1–S4). Approximately half of all considered studies presented here were conducted in Europe, independently of the type of infections with Crypto. Figure 1 shows that the highest mean prevalence of Crypto-BRV was detected in Ireland (16.7%) and the highest mean prevalence of Crypto-BCoV was presented in the United Kingdom (4.3%) and Germany (4.3%), whilst the highest mean prevalence of Crypto-ETEC was identified in Turkey (4.8%). Table 1 shows that the factor “geographical region” was influential on the prevalence level of Crypto, Crypto-BRV and Crypto-ETEC and could explain between 11.4% and 23.6% of the variance (i.e., R^2^ = proportion of variance) in the published worldwide prevalence in the calf population.

**Figure 2:**
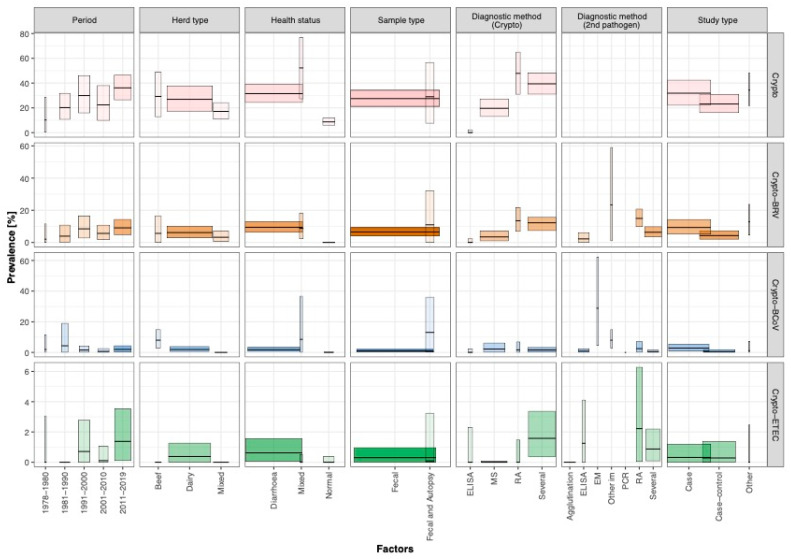
Crypto prevalence and associated concurrent infections presented as box plot and stratified by influencing factors (i.e., period, herd type, health status, sample type, diagnostic method, and study type). One colour is assigned to a specific Crypto infection. The darker the colour within a colour range, the more animals were included in the respective factor. The wider the box plots are, the more (sub)-studies were considered in the respective factor. N.B. A detailed description of all analysed factors can be found in Tables S1–S4.

Figure 2 shows the prevalence of Crypto across the studies stratified by analysed influencing factors. The factor “period” was determined as a significant influencing factor for the level of prevalence of Crypto-ETEC (Table 1). No significant difference regarding the estimated mean prevalence was observed for Crypto-BCoV mixed infections during the period (Figure 2 and Table 1).

The factor “health status of sampled animals” and “diagnostic” significantly influenced the prevalence of Crypto and associated mixed infections, except for Crypto-ETEC. The prevalence of concurrent infections with Crypto for each study and their weight contribution proportion to the meta-analyses stratified by the health status of the calves is shown in Figures S2–S5. The chance of detecting BRV, BCoV, and ETEC in calves with diarrhoea was 0.8 (CI: 0.6–1.0), 0.7 (CI: 0.5–1.0), and 0.6 (CI: 0.4–0.9) lower in the presence of Crypto, compared to calves without Crypto. 

Figure 2 shows that the prevalence of Crypto and Crypto-BRV was more than twice and fourfold lower, respectively, when using microscopy (MS) as a diagnostic method (Crypto: 19.6%; CI: 12.9–27.3; Crypto-BRV: 3.4; CI: 0.8–7.2) compared to lateral flow immunochromatographic assay (RA) (Crypto: 47.9%; CI: 30.8–65.1; Crypto-BRV: 13.5; CI: 6.8–21.7) across all studies. In general, prevalence was lower when using faecal samples rather than faecal and autopsy samples together, with the exception of Crypto-ETEC. As an example, the prevalence of Crypto-BCoV was 13 times lower when using faecal samples instead of analysing faecal and autopsy samples together (see Figure 2 and Tables S1–S4). Overall, the proportion of variance in the level of the prevalence of Crypto and associated concurrent infections with Crypto can mainly be explained by the “diagnostic” factor (R^2^ min–max: 0.0–40.3%), followed by the “health status of the sampled animals” (R^2^ min–max: 1.4–27.3%) and “geographical region“ (R^2^ min–max: 5.9–23.6%; (Table 1)).

**Figure 3:**
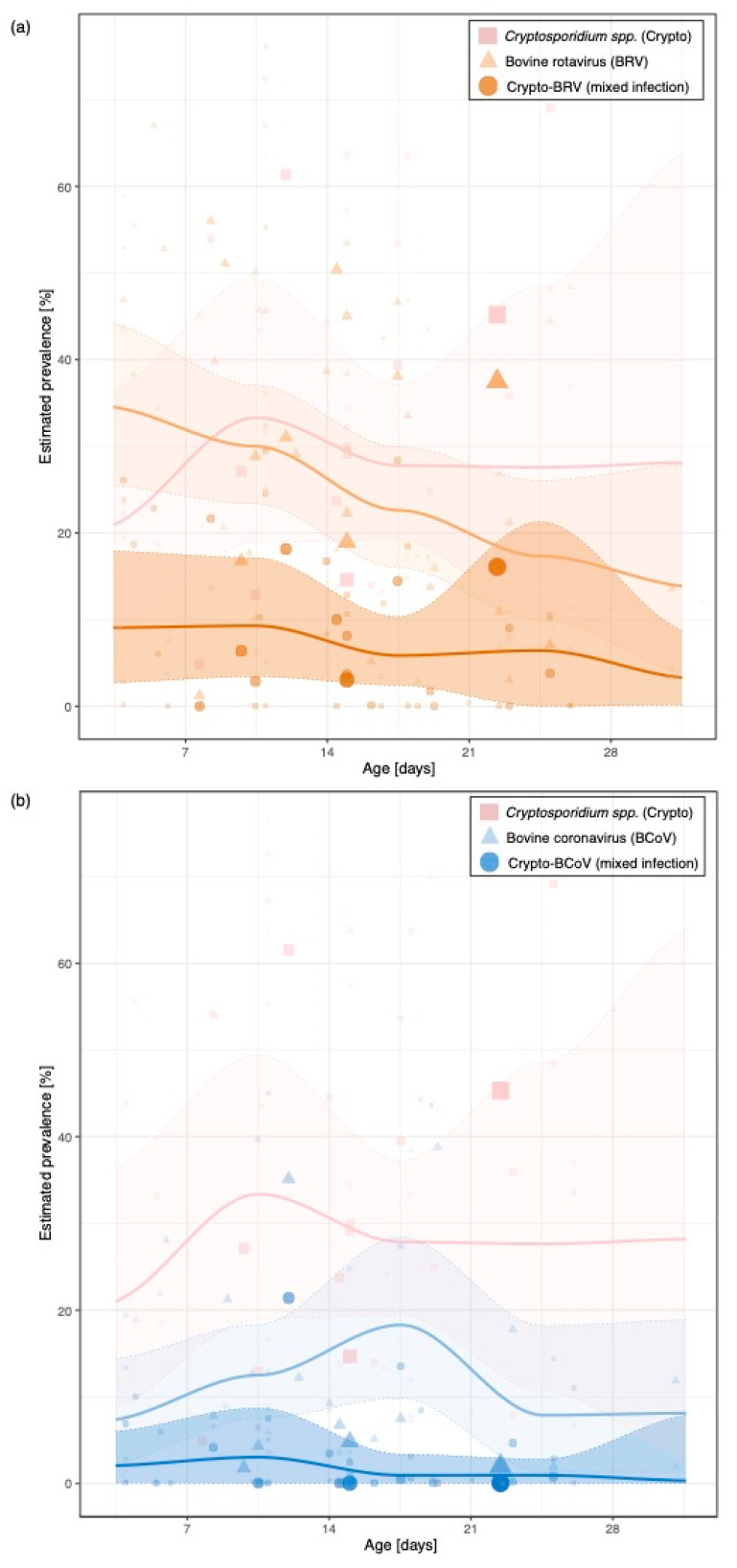

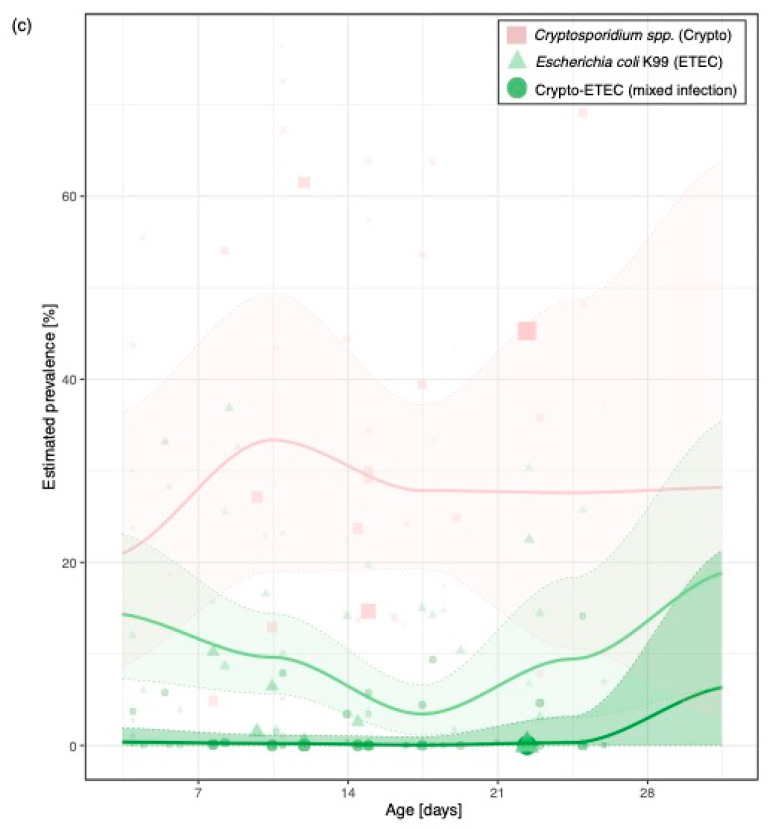
*Cryptosporidium* spp. (Crypto), (**a**) bovine rotavirus (BRV), (**b**) bovine coronavirus (BCoV), (**c**) *Escherichia coli* F5 (K99) (ETEC) prevalence and associated mixed infection with Crypto depending on the age of sampled animals until 30 days. The mean prevalence estimates of all incorporated studies are represented as lines with the corresponding 95% CI (area) and individual prevalence values of the studies during the period observed are presented as dot symbols. The wider the dot symbols are the more prevalence estimates at a certain age group of sampled animals are available. N.B. Studies in the age groups (35–49 days; see Table S1–S4) were excluded from the figure here to avoid imprecise predictions because only small number of available studies were available in this age group (see number of available studies in each age groups in the Tables S1–S4).

Figure 3 shows the prevalence of Crypto and associated mixed infections with BRV, BCoV, and ETEC (Figure 3a–c) depending on the age of sampled animals until 30 days. While the courses of the Crypto-BRV and Crypto-BCoV mixed infection are similar and the peak in prevalence of both occurs between 7 and 14 days of animal age (Crypto-BRV: 9.3%, CI: 3.4–17.1; Crypto-BCoV: 3.0%; CI: 0.0–8.7), the course differs from that of the Crypto-ETEC mixed infection, where the highest prevalence occurs after 28 days of animal age (Crypto-ETEC: 6.3%, CI: 0.0–21.2 (see Figure 3 and Tables S1–S4)). 

## 4. Discussion

The assessment of the prevalence of Crypto related to the geographical distribution and the influencing factors in combination with other enteric pathogens in the calf population is of particular importance to (veterinary) public authorities as it can increase the visibility of the worldwide (heterogeneous) distribution of Crypto mixed infections, and thus, support policy makers in the decision-making process, if testing and mitigation measures, as well as further epidemiological studies are required.

Multiple enteric agents, parasitic (e.g., *Cryptosporidium parvum*, *Eimeria* spp.), viral (e.g., bovine coronavirus, bovine rotavirus, bovine viral diarrhoea virus (BVDV)), and bacterial (e.g., *Escherichia coli* F5 (K99), *Salmonella* spp.) are causative pathogens of neonatal calf diarrhoea [14,15,16,17,18,19,20,21]. The study presented here is based on the genus level of *Cryptosporidium* spp. because studies used other diagnostic methods, like acid-fast staining, which do not allow a differentiation of the species [9,22]. All potential influencing factors analysed in the presented study could explain more than half of the variance (on average R^2^ = 51.6%) in the level of the worldwide prevalence of Crypto and associated mixed-infection across the studies, whereby the factor “diagnostic” explained half of this variance (Table 1). The study by Brunauer and colleagues shows a similar impact of the factor “diagnostic” on the level of the prevalence of BRV and associated mixed-infection [9]. Other meta-analysis studies could not identify a significant impact of the “diagnostic” factor on the reported prevalence of pathogens such as BVDV [8]. Besides the factor “diagnostic” (R^2^ min–max: 0.0–40.3%), the “health status of sampled animals” (R^2^ min–max: 1.4–27.3%), and the “geographical region” (R^2^ min–max: 5.9–23.6%) were both identified as influential factors with a high proportion of variance to explain the different prevalence levels of Crypto across the studies. The latter stated in contrast to the study by Brunauer et al., where the geographical region had a very low explanatory power. In general, the influencing factors analysed here better explained the different levels of Crypto mixed infections across the literature (see: R^2^ of the reduced model in Table 1) than those analysed by Brunauer for BRV mixed infections. Further, Crypto mixed infections were 2–6 times (Crypto-BCoV: 1.4%; Crypto-ETEC: 0.3%) less common compared to BRV concurrent infections (BRV-BCoV: 2.8%; BRV-ETEC: 1.6%), as presented in the study by Brunauer [9].

The other factors analysed in the study presented here such as “sample type” and “study type” differ greatly in their explanatory power and/or significance level on the worldwide prevalence of Crypto (Table 1). The heterogeneous distribution of the influencing factors and the proportion of variance (R^2^) could be explained by the fact that the four pathogens considered in the study presented here differ in their virulence, infectivity, pathogenicity, and environmental resistance [9,23]. The chance of detecting BRV, BCoV, and ETEC in calves with diarrhoea was 0.8 (CI: 0.6–1.0), 0.7 (CI: 0.5–1.0) and 0.6 (CI: 0.4–0.9) lower in the presence of Crypto, compared to calves without Crypto. This may indicate an inhibitory effect between BRV, BCoV, ETEC, and Crypto in calves. 

The analysis presented here revealed a wide variation in the prevalence of concurrent infection with Crypto within (Tables S2–S4) and between geographical regions (Figure 1), and exposed an incomplete picture of the epidemiological situation of Crypto mixed infections for a number of regions (see Figure 1). The latter might be the result of testing activities not being performed due to a low number of calves, and/or prevalence not being reported, and/or prevalence data being available but not in the public domain, and/or these pathogens having a low priority level for being controlled; Crypto is a parasite with zoonotic potential and should receive attention in the routine testing schemes of veterinary authorities. Furthermore, as discussed in other studies [9,18], the “geographical region” incorporated several other factors such as the husbandry system, general cattle herd structure, law standards, and biosecurity standards, all of which can influence the prevalence level of Crypto and associated concurrent infection with Crypto in the calf population. We assume that this might explain the heterogeneous distribution in the prevalence of Crypto between the countries (Figure 1). To increase the proportion of variance in the study presented here it is desirable to incorporate and analyse further factors like the meta-data of the livestock structure per region, the presence of antibodies through colostral consumption in the first week of animal life, and/or other management-related factors such as the separation of animals based on age and/or a hygienic score of the sampled farms and further environmental factors such as the season (e.g., calves born in winter have an increased risk for diarrhoea) in order to explain the different levels of the prevalence across the literature [24,25,26,27,28,29]. In the study presented here these factors could not be analysed due to a lack of data in the analysed studies. Figure 2 indicated that the analysis of prevalence in dependency of influencing factors is essential, because the prevalence can vary significantly for each analysed factor. Consequently, the heterogeneity of the prevalence of Crypto can be better explained by taking into account various influencing factors, instead of just depicting a pooled prevalence.

Figure 3 shows that the prevalence of Crypto-ETEC infections is 0.3% lower than other mixed-infections with Crypto, which can be explained by the fact that ETEC-caused diarrhoea in calves usually occurs in the first four days of animal age [1] and Crypto oocysts are not excreted until three days of animal age [30]. A common occurrence in older calves is likely, as both ETEC and Crypto can be present in the digestive tract of these animals without causing any clinical symptoms [1,15]. However, this increase may also be under- and/or overestimated due to the small number of studies within the older age groups of sampled animals (Tables S1–S4). Furthermore, there is a significant correlation between study size and Crypto-ETEC prevalence, which is the consequence of the overall low prevalence, as rare animal diseases would need larger samples to be reliably detected. The overall number of mixed Crypto-BCoV infections with an estimated mean prevalence of 1.4% is relatively low. The peak, with a prevalence of approximately 3%, is reached between 7 and 10 days of animal age and decreases steadily from this point on. The identified peak of prevalence in the study presented here is confirmed in other studies [31]. In general, the identified course of concurrent infection with Crypto related to the age of sampled animals is confirmed in several studies analysing the prevalence depending on age of animals [5,32,33].

Our study provides relevant information about the global distribution, global frequency of the concurrent infection with Crypto and its influencing factors which can support decision-makers in relation to the burden of zoonotic pathogens in the animal population. Furthermore, the study results indicated an inhibitory effect between BRV, BCoV, ETEC and Crypto and identified gaps related to nonreported concurrent infection with Crypto in several countries. Overall, this emphasizes the need for more standardised epidemiological studies in order to interpret the results more accurately. In particular, reports and analyses of further potential influencing factors on the prevalence of Crypto in the calf population (e.g., the season, hygiene level at the farms) would increase our understanding of the transmission of (zoonotic) agents and support the implementation of prevention and intervention measures to reduce the prevalence of Crypto in the animal population.

## 5. Conclusions

As far as the authors are aware, the study presented here provides the first worldwide meta-analysis regarding concurrent infections with Crypto. The study results revealed (i) a heterogeneous distribution of concurrent infection with Crypto between countries, (ii) an inhibitory effect between BRV, BCoV, ETEC, and Crypto in calves and (iii) a high variance in prevalence across the studies which is mainly explained by the influencing factors “diagnostic”, “health status of the sampled animals”, and the “geographical region”.

## Figures and Tables

**Table 1:** Uni- and multivariate meta-regression analysis stratified by different types of Crypto infection and potential influencing factors.

Univariate (Crypto)			Univariate (Crypto-BRV)			Univariate (Crypto-BCoV)			Univariate (Crypto-ETEC)		
Factors	R^2^	*p* ^a^	Factors	R^2^	*p* ^a^	Factors	R^2^	*p* ^a^	Factors	R^2^	*p* ^a^
Region	11.4	0.1 *	Region	12.0	0.1 *	Region	5.9	0.2	Region	23.6	0.0 *
Period	2.3	0.3 *	Period	0.0	0.5	Period	0.0	0.9	Period	14.7	0.0 *
Number of herds	0.0	0.7	Number of herds	0.0	0.6	Number of herds	0.0	1.0	Number of herds	0.0	0.9
Herd type	3.1	0.2 *	Herd type	4.3	0.2 *	Herd type	6.5	0.2 *	Herd type	16.1	0.0 *
Age class	0.0	0.8	Age class	0.0	0.5	Age class	0.0	0.8	Age class	5.1	0.2 *
Health status	20.7	0.0 **	Health status	27.3	<0.0 **	Health status	17.1	0.0 **	Health status	1.4	0.3
Sample size	0.0	0.7	Sample size	0.0	0.7	Sample size	0.0	0.4	Sample size	15.9	0.0 **
Sample type	0.0	0.9	Sample type	0.0	0.6	Sample type	13.1	0.0 **	Sample type	0.0	0.9
Diagnostic Crypto	40.3	0.0 **	Diagnostic Crypto	22.8	<0.0 *	Diagnostic Crypto	0.0	0.8	Diagnostic Crypto	20.7	0.0 **
-	-	-	Diagnostic BRV	22.5	<0.0 **	Diagnostic BCoV	39.0	<0.0 **	Diagnostic ETEC	26.2	0.0 **
Study type	0.0	0.4	Study type	4.5	0.1 *	Study type	5.1	0.2 *	Study type	0.0	0.8
**Multivariate (Crypto)**			**Multivariate (Crypto-BRV)**			**Multivariate (Crypto-BCoV)**			**Multivariate (Crypto-ETEC)**		
**Number of Factors**	**R^2^**	**AICc ^b^**	**Number of Factors**	**R^2^**	**AICc ^b^**	**Number of Factors**	**R^2^**	**AICc ^b^**	**Number of Factors**	**R^2^**	**AICc ^b^**
Full Model (*n* = 5; *p* < 0.25 *)	59.0	15.4	Full Model (*n* = 6; *p* < 0.25 *)	46.2	–4.6	Full Model (*n* = 5; *p* < 0.25 *)	53.9	–45.9	Full Model (*n* = 7; *p* < 0.25 *)	47.3	–7.2
Reduced Model (*n* = 2 **)	60.7	−23.6	Reduced Model (*n* = 2 **)	49.5	–48.2	Reduced Model (*n* = 3 **)	49.8	–55.1	Reduced Model (*n* = 3 **)	58.3	–93.7

R^2^ = R-squared is a goodness-of-fit measure for regression models and expresses the proportion of variance in the dependent variable that can be explained by the independent variable. ^a^ = Factors with a *p*-value cut-off point lower than 0.25 from the univariate regression analysis were also considered in the multivariate regression analysis. A detail description of the meta-regression analysis is published by Brunauer et al. [9]. ^b^ = AICc—Akaike information criterion in order to select the most important factors in the model without declining the model-fit accuracy [10], corrected for small sample size. * = considered in the full model and ** in the reduced model after factor selection via AICc.

## Data Availability

The data presented in this study are available in the supplementary material. The digital datasets and analyses of the present study are available from the corresponding author upon request.

## Supplementary Materials

The following are available online at https://www.mdpi.com/article/10.3390/ani11061786/s1. Table S1: Subgroup meta-analysis of studies reporting the prevalence of *Cryptosporidium* spp. (Crypto); Table S2: Subgroup meta-analysis of studies reporting the prevalence of *Cryptosporidium* spp. (Crypto) and Bovine rotavirus (BRV); Table S3: Subgroup meta-analysis of studies reporting the prevalence of *Cryptosporidium* spp. (Crypto) and Bovine Coronavirus (BCoV); Table S4: Subgroup meta-analysis of studies reporting the prevalence of *Cryptosporidium* spp. (Crypto) and *Escherichia coli* F5 (K99) (ETEC); Table S5: Estimated mean prevalence (%) of the pathogens in the various countries, shown in Figure 1. Figure S1: Studies incorporated in the presented study; Figure S2: Forest plot of prevalence with *Cryptosporidium* spp. (Crypto) ordered by health status and publication year of the studies; Figure S3: Forest plot of prevalence with *Cryptosporidium* spp. (Crypto) and bovine rotavirus (BRV) ordered by health status and publication year of the studies; Figure S4: Forest plot of prevalence with *Cryptosporidium* spp. (Crypto) and bovine coronavirus (BCoV) ordered by health status and publication year of the studies; Figure S5: Forest plot of prevalence with *Cryptosporidium* spp. (Crypto) and *Escherichia coli* F5 (K99) (ETEC) ordered by health status and publication year of the studies; Figure S6: Case influence diagnostic analysis (a) and funnel plot (b) of *Cryptosporidium* spp. (Crypto) and the identified outliers (shown as red circles). N.B. In case of Crypto-BRV no outliers were identified. The regression test for funnel plot asymmetry in the meta-analysis indicated no publication bias for Crypto (z = −0.33; *p* = 0.73); Figure S7: Case influence diagnostic analysis (a) and funnel plot (b) of *Cryptosporidium* spp. (Crypto) and bovine rotavirus (BRV) and the identified outliers (shown as red circles). N.B. In case of Crypto-BRV no outliers were identified. The regression test for funnel plot asymmetry in the meta-analysis indicated no publication bias for Crypto-BRV (z = −0.25; *p* = 0.79); Figure S8: Case influence diagnostic analysis (a) and funnel plot (b) of *Cryptosporidium* spp. (Crypto) and bovine coronavirus (BCoV) and the identified outliers (shown as red circles). The regression test for funnel plot asymmetry in the meta-analysis indicated no publication bias for Crypto-BCoV (z = 1.89; *p* = 0.05); Figure S9: Case influence diagnostic analysis (a) and funnel plot (b) of *Cryptosporidium* spp. (Crypto) and *Escherichia coli* F5 (K99) (ETEC) and the identified outliers (shown as red circles). The regression test for funnel plot asymmetry in the meta-analysis indicated a publication bias for Crypto-ETEC (z = 2.30; *p* = 0.02).
