# Supplementary material for: Cryptosporidium spp. Infections in Combination with Other Enteric Pathogens in the Global Calf Population
Source: Animals (Basel). 2021 Jun 15;11(6):1786. doi: 10.3390/ani11061786 (PMC8232747; doi:10.3390/ani11061786)

SUPPLEMENTARY MATERIAL

Table S1. Subgroup meta-analysis of studies reporting the prevalence of *Cryptosporidium spp.* (Crypto).

Crypto							
	**Sample Size (No. Animals)**	**No. Studies**	**No. Prevalence Inputs**	**Weighted Mean Estimate (%)**	**Confidence Interval (95%)**	**Qep[9]**	**I2 (%)[10]**
Overall	7191	28	56	27.57	(21.45-34.10)	<0.01	96.70
**Geographical region[1]**							
Europe	4235	16	26	34.10	(25.27-43.48)	<0.01	96.82
North America	240	3	6	25.58	(9.72-45.33)	<0.01	88.97
South America	452	1	2	29.44	(25.15-33.90)	0.85	0.00
Africa	328	2	3	9.38	(0.00-38.58)	<0.01	97.46
East Asia	251	1	2	19.23	(9.64-30.86)	0.04	76.80
South Asia	193	2	9	14.61	(2.78-32.14)	<0.01	86.58
West Asia	266	2	6	44.57	(26.92-62.90)	<0.01	87.88
Oceania	1226	1	2	8.98	(1.31-21.01)	<0.01	96.65
**Period[2]**							
1978-1980	59	1	2	10.23	(0.24-28.57)	0.08	68.03
1981-1990	1465	5	11	20.22	(10.54-31.84)	<0.01	94.86
1991-2000	820	6	12	29.91	(15.78-46.13)	<0.01	95.11
2001-2010	1313	8	13	22.42	(9.71-38.14)	<0.01	96.91
2011-2019	3534	8	18	36.15	(26.3-46.57)	<0.01	96.44
**Herd type[3]**							
Dairy	3892	16	29	26.90	(17.17-37.80)	<0.01	97.80
Beef	304	2	4	29.25	(12.69-48.98)	<0.01	86.17
Mixed	895	5	10	17.13	(10.83-24.38)	<0.01	79.87
Not specified	2100	6	13	38.08	(28.19-48.46)	<0.01	91.78
**Age class (in days)[4]**							
0-7	690	8	9	20.90	(8.70-36.22)	<0.01	91.42
7-14	1943	12	15	33.26	(18.91-49.27)	<0.01	97.66
14-21	2637	15	20	27.76	(19.26-37.08)	<0.01	95.33
21-28	1843	7	8	27.53	(10.55-48.39)	<0.01	97.83
28-35	28	2	2	28.09	(3.06-63.61)	0.06	71.07
35-42	-	-	-	-	-	-	-
42-49	50	2	2	13.84	(0.00-57.94)	<0.01	87.48
**Health status[5]**							
Diarrhoea	4269	24	42	31.51	(24.22-39.25)	<0.01	95.54
Normal	664	9	9	8.68	(5.87-11.89)	0.14	25.45
Mixed	1032	3	3	52.26	(27.09-76.88)	<0.01	98.45
Not specified	1226	1	2	8.98	(1.31-21.01)	<0.01	96.65

**Sample type**[6]							
Fecal	7111	26	51	27.49	(21.13-34.29)	<0.01	96.96
Both (Fecal+Autopsy)	80	2	5	28.96	(7.39-56.74)	<0.01	81.50
**Diagnostic method (Crypto)**[7]							
ELISA	93	1	4	<0.01	(0.00-2.39)	0.97	0.00
RA	1794	4	4	47.86	(30.83-65.14)	<0.01	96.50
Several	2957	11	24	39.41	(30.93-48.21)	<0.01	94.55
MS	2347	12	24	19.62	(12.88-27.27)	<0.01	93.69
**Study type**[8]							
Case-control	4109	13	26	23.12	(16.05-30.97)	<0.01	96.32
Case	3030	14	29	31.84	(21.96-42.56)	<0.01	96.33
Other	52	1	1	34.34	(21.70-48.12)	1.00	0.00

[1] Geographical region = Individual countries were assigned to Europe, Australia, West Asia, East Asia, South Asia, North America, South America and Africa.
[2] Period = Sample period described the begin and end of sampling in the studies. In case that the sampling period was not reported the publication data was used.
[3] Herd type = More than one herd types was assigned to the category "mixed" and the category "not specified" included studies, which not defined the herd type.
[4] Age class = If available in the studies, the median or mean of the animal age was used. If the median or mean of the animal age was not available, the center of the published age range was calculated and were clustered in 7-day periods.
[5] Health status = The health status cover animals with diarrhoea, normal (=without diarrhoea), mixed (=both diarrhoea and normal) and studies without information to the health status were assigned in the category "not specified".
[6] Sample type = This category included fecal samples or fecal and autopsy samples.
[7] Diagnostic method = This category covered ELISA= Antigen enzyme-linked immunosorbent assay; RA= Lateral flow immunochromatographic assay; Several= covered more than one diagnostic methods for screening and confirmation of laboratory results; MS= Acid-fast staining.
[8] Study type = Case-control studies covered studies which tested diarrheic and normal calves and/or studies sampled animals in several regions of a country; Case studies incorporated studies which tested animals with diarrhoea and/or and/or studies sampled animals in several regions of a country.
[9] Qep = The Q statistic and its p-value serve as a test of significance.
[10] I^2 = The ratio of true heterogeneity to total variation in calculated effects, expressed in %.

Table S2. Subgroup meta-analysis of studies reporting the prevalence of *Cryptosporidium spp.* (Crypto) and Bovine rotavirus (BRV).

Crypto-BRV							
	Sample Size (No. Animals)	No. Studies	No. Prevalence Inputs	Weighted Mean Estimate (%)	Confidence Interval (95%)	Qep[9]	I2 (%)[10]
Overall	7191	28	56	6.69	(4.27-9.51)	<0.01	92.55
**Geographical region[1]**							
Europe	4235	16	26	8.90	(4.98-13.65)	<0.01	94.03
North America	240	3	6	6.59	(0.21-18.16)	<0.01	83.92
South America	452	1	2	5.68	(1.99-10.67)	0.07	69.09
East Asia	251	1	2	0.79	(0.00-3.08)	0.28	15.43
West Asia	266	2	6	16.61	(8.03-27.19)	0.01	73.82
South Asia	193	2	9	1.98	(0.00-6.76)	0.03	48.39
Oceania	1226	1	2	2.27	(0.91-4.05)	0.14	52.98
Africa	328	2	3	0.62	(0.00-8.81)	<0.01	91.29
**Period[2]**							
1978-1980	59	1	2	2.04	(0.00-11.65)	0.16	49.67
1981-1990	1465	5	11	3.92	(0.10-10.93)	<0.01	94.44
1991-2000	820	6	12	8.46	(2.66-16.47)	<0.01	89.89
2001-2010	1313	8	13	5.62	(1.79-10.88)	<0.01	88.07
2011-2019	3534	8	18	9.07	(4.72-14.44)	<0.01	93.35
**Herd type[3]**							
Dairy	3892	16	29	6.13	(2.90-10.19)	<0.01	93.46
Beef	304	2	4	5.64	(0.03-16.64)	0.01	80.25
Mixed	895	5	10	3.23	(0.61-7.17)	<0.01	75.81
Not specified	2100	6	13	12.37	(6.04-20.24)	<0.01	91.71
**Age class (in days)[4]**							
0-7	690	8	9	9.04	(2.72-17.87)	<0.01	83.75
7-14	1943	12	15	9.27	(3.42-17.10)	<0.01	94.83
14-21	2637	15	20	5.84	(2.41-10.29)	<0.01	92.00
21-28	1843	7	8	3.28	(0.16-8.74)	<0.01	90.48
28-35	28	2	2	6.41	(0.00-21.29)	0.26	20.31
35-42	-	-	-	-	-	-	-
42-49	50	2	2	7.80	(0.00-27.97)	0.11	60.99
**Health status[5]**							
Diarrhoea	4269	24	42	9.43	(6.28-13.06)	<0.01	89.62
Normal	664	9	9	0.00	(0.00-0.03)	0.60	0.00
Mixed	1032	3	3	8.78	(2.19-18.29)	<0.01	94.30
Not specified	1226	1	2	2.27	(0.91-4.05)	0.14	52.98
**Sample type[6]**							
Fecal	7111	26	51	6.50	(4.09-9.32)	<0.01	92.72

Both (Fecal+Autopsy)	80	2	5	10.96	(0.04-32.23)	0.00	77.94
**Diagnostic method (Crypto)**[7]							
ELISA	93	1	4	<0.01	(0.00-2.39)	0.97	0.00
RA	1794	4	4	13.49	(6.80-21.74)	<0.01	89.72
Several	2957	11	24	12.21	(7.27-15.75)	<0.01	89.01
MS	2347	12	24	3.44	(0.85-7.16)	<0.01	91.00
**Diagnostic method (BRV)**[7]							
ELISA	1086	7	16	2.21	(0.06-6.16)	<0.01	84.92
RA	2412	6	10	14.94	(9.76-20.89)	<0.01	87.27
Several	3672	14	27	6.33	(3.40-9.91)	<0.01	91.30
Other im	21	1	3	23.33	(0.97-59.14)	0.05	65.92
**Study type**[8]							
Case-control	4109	13	26	4.27	(1.89-7.30)	<0.01	91.55
Case	3030	14	29	9.29	(5.13-14.34)	<0.01	91.39
Other	52	1	1	12.82	(4.59-23.81)	1.00	0.00

[1] Geographical region = Individual countries were assigned to Europe, Australia, West Asia, East Asia, South Asia, North America, South America and Africa.
[2] Period = Sample period described the begin and end of sampling in the studies. In case that the sampling period was not reported the publication data was used.
[3] Herd type = More than one herd types was assigned to the category “mixed” and the category “not specified” included studies, which not defined the herd type.
[4] Age class = If available in the studies, the median or mean of the animal age was used. If the median or mean of the animal age was not available, the center of the published age range was calculated and were clustered in 7-day periods.
[5] Health status = The health status cover animals with diarrhoea, normal (=without diarrhoea), mixed (=both diarrhoea and normal) and studies without information to the health status were assigned in the category “not specified”.
[6] Sample type = This category included fecal samples or fecal and autopsy samples.
[7] Diagnostic method = This category covered ELISA= Antigen enzyme-linked immunosorbent assay; RA= Lateral flow immunochromatographic assay; Several= covered more than one diagnostic methods for screening and confirmation of laboratory results; MS= Acid-fast staining; Other im = Other antibody-based methods besides ELISA and RA (i.e., Immunohistochemical / immunostaining, Fluorescence antibody technique).
[8] Study type = Case-control studies covered studies which tested diarrheic and normal calves and/or studies sampled animals in several regions of a country; Case studies incorporated studies which tested animals with diarrhoea and/or and/or studies sampled animals in several regions of a country.
[9] Qep = The Q statistic and its p-value serve as a test of significance.
[10] I^2 = The ratio of true heterogeneity to total variation in calculated effects, expressed in %.

Table S3. Subgroup meta-analysis of studies reporting the prevalence of *Cryptosporidium spp.* (Crypto) and Bovine Coronavirus (BCoV).

Crypto-BCoV							
	**Sample Size (No. Animals)**	**No. Studies**	**No. Prevalence Inputs**	**Weighted Mean Estimate (%)**	**Confidence Interval (95%)**	**Qep[9]**	**I2 (%)[10]**
Overall	5973	24	49	1.45	(0.44-2.83)	<0.01	84.71
**Geographical region[1]**							
Europe	3469	13	21	1.79	(0.07-4.88)	<0.01	92.88
North America	240	3	6	6.75	(2.43-12.54)	0.10	44.51
South America	-	-	-	-	-	-	-
Africa	328	2	3	0.51	(0.00-4.92)	<0.01	79.03
East Asia	251	1	2	0.00	(0.00-0.02)	0.95	0.00
South Asia	193	2	9	0.93	(0.00-3.55)	0.40	4.50
West Asia	266	2	6	1.74	(0.04-4.87)	0.17	29.86
Oceania	1226	1	2	0.00	(0.00-0.12)	0.99	0.00
**Period[2]**							
1978-1980	59	1	2	1.94	(0.00-11.55)	0.16	49.67
1981-1990	734	3	7	4.34	(0.00-19.13)	<0.01	95.97
1991-2000	757	5	10	1.59	(0.07-4.29)	<0.01	66.13
2001-2010	889	7	12	0.65	(0.00-2.65)	<0.01	65.41
2011-2019	3534	8	18	1.97	(0.32-4.49)	<0.01	87.72
**Herd type[3]**							
Dairy	2950	13	25	1.92	(0.48-3.97)	<0.01	82.16
Beef	91	1	3	7.99	(2.79-15.01)	0.53	0.00
Mixed	832	4	8	0.00	(0.00-0.15)	0.61	0.00
Not specified	2100	6	13	1.99	(0.00-7.09)	<0.01	92.83
**Age class (in days)[4]**							
0-7	690	8	9	2.01	(0.06-5.60)	<0.01	60.29
7-14	1240	10	12	2.99	(0.03-8.65)	<0.01	91.19
14-21	2122	13	16	0.90	(0.00-3.32)	<0.01	86.89
21-28	1843	7	8	0.91	(<0.01-2.75)	0.02	61.34
28-35	28	2	2	0.28	(0.00-7.81)	0.85	0.00
35-42	-	-	-	-	-	-	-
42-49	50	2	2	0.00	(0.00-3.62)	0.69	0.00
**Health status[5]**							
Diarrhoea	3764	21	38	1.80	(0.57-3.48)	<0.01	79.75
Normal	375	7	7	0.00	(0.00-0.34)	0.96	0.00
Mixed	608	2	2	8.54	(0.00-36.87)	<0.01	98.39
Not specified	1226	1	2	0.00	(0.00-0.12)	0.99	0.00
**Sample type[6]**							
Fecal	5893	22	44	1.09	(0.29-2.21)	<0.01	80.54

Both (Fecal+Autopsy)	80	2	5	13.09	(0.29-36.21)	<0.01	79.51
**Diagnostic method (Crypto)**[7]							
ELISA	93	1	4	0.00	(0.00-2.30)	0.97	0.00
RA	1370	3	3	1.17	(0.00-7.01)	0.01	77.05
Several	2957	11	24	1.61	(0.38-3.39)	<0.01	78.38
MS	1553	9	18	2.21	(0.04-6.30)	<0.01	90.33
**Diagnostic method (BCoV)**[7]							
ELISA	2259	8	16	0.83	(0.02-2.41)	<0.01	74.79
RA	1988	5	9	2.48	(0.03-7.13)	<0.01	89.60
Several	1363	8	16	0.48	(0.00-1.65)	0.02	45.45
EM	91	1	3	7.99	(2.79-15.01)	0.53	0.00
Other im	21	1	3	28.88	(4.41-62.22)	0.11	59.19
PCR	251	1	2	0.00	(0.00-0.02)	0.95	0.00
**Study type**[8]							
Case-control	3343	10	21	0.48	(0.00-1.88)	<0.01	84.64
Case	2578	13	27	2.83	(0.88-5.52)	<0.01	81.02
Other	52	1	1	1.17	(0.00-7.26)	1.00	0.00

[1] Geographical region = Individual countries were assigned to Europe, Australia, West Asia, East Asia, South Asia, North America, South America and Africa.
[2] Period = Sample period described the begin and end of sampling in the studies. In case that the sampling period was not reported the publication data was used.
[3] Herd type = More than one herd types was assigned to the category "mixed" and the category "not specified" included studies, which not defined the herd type.
[4] Age class = If available in the studies, the median or mean of the animal age was used. If the median or mean of the animal age was not available, the center of the published age range was calculated and were clustered in 7-day periods.
[5] Health status = The health status cover animals with diarrhoea, normal (=without diarrhoea), mixed (=both diarrhoea and normal) and studies without information to the health status were assigned in the category "not specified".
[6] Sample type = This category included fecal samples or fecal and autopsy samples.
[7] Diagnostic method = This category covered ELISA= Antigen enzyme-linked immunosorbent assay; RA= Lateral flow immunochromatographic assay; Several= covered more than one diagnostic methods for screening and confirmation of laboratory results; MS= Acid-fast staining; EM= Electron microscopy; Other im = Other antibody-based methods besides ELISA and RA (i.e., Immunohistochemical / immunostaining, Fluorescence antibody technique); PCR=Polymerase chain reaction.
[8] Study type = Case-control studies covered studies which tested diarrheic and normal calves and/or studies sampled animals in several regions of a country; Case studies incorporated studies which tested animals with diarrhoea and/or and/or studies sampled animals in several regions of a country.
[9] Qep = The Q statistic and its p-value serve as a test of significance.
[10] I^2 = The ratio of true heterogeneity to total variation in calculated effects, expressed in %.

Table S4. Subgroup meta-analysis of studies reporting the prevalence of *Cryptosporidium spp.* (Crypto) and *Escherichia coli* F5 (K99) (ETEC).

Crypto-ETEC							
	**Sample Size (No. Animals)**	**No. Studies**	**No. Prevalence Inputs**	**Weighted Mean Estimate (%)**	**Confidence Interval (95%)**	**Qep[9]**	**I2 (%)[10]**
Overall	5719	26	52	0.28	(0.00-0.88)	<0.01	67.39
**Geographical region[1]**							
Europe	3811	15	25	0.09	(0.00-0.75)	<0.01	69.04
North America	240	3	6	0.56	(0.00-2.73)	0.30	10.08
South America	452	1	2	0.00	(0.00-0.00)	0.97	0.00
Africa	328	2	3	0.12	(0.00-4.55)	<0.01	83.63
East Asia	-	-	-	-	-	-	-
South Asia	193	2	9	0.61	(0.00-2.90)	0.71	0.00
West Asia	266	2	6	4.71	(0.69-10.99)	<0.01	65.16
Oceania	429	1	1	0.00	(0.00-0.19)	1.00	0.00
**Period[2]**							
1978-1980	59	1	2	0.00	(0.00-3.01)	0.95	0.00
1981-1990	1465	5	11	0.00	(0.00-0.00)	0.62	0.00
1991-2000	820	6	12	0.71	(0.00-2.79)	<0.01	65.00
2001-2010	638	6	10	0.11	(0.00-1.07)	0.16	18.22
2011-2019	2737	8	17	1.38	(0.10-3.54)	<0.01	80.78
**Herd type[3]**							
Dairy	2671	15	27	0.38	(0.00-1.28)	<0.01	62.22
Beef	304	2	4	0.00	(0.00-0.02)	0.82	0.00
Mixed	895	5	10	0.00	(0.00-0.00)	0.80	0.00
Not specified	1849	5	11	2.30	(0.16-5.92)	<0.01	78.88
**Age class (in days)[4]**							
0-7	690	8	9	0.37	(0.00-1.88)	0.11	24.10
7-14	1519	11	14	0.21	(0.00-1.13)	0.02	45.37
14-21	1589	13	17	0.04	(0.00-0.93)	<0.01	67.00
21-28	1843	7	8	0.30	(0.00-3.16)	<0.01	87.42
28-35	28	2	2	6.31	(0.00-21.22)	0.26	20.31
35-42	-	-	-	-	-	-	-
42-49	50	2	2	5.24	(0.26-13.97)	0.36	0.00
**Health status[5]**							
Diarrhoea	4132	23	41	0.62	(0.06-1.57)	<0.01	69.59
Normal	550	8	8	0.00	(0.00-0.39)	0.97	0.00
Mixed	608	2	2	0.00	(0.00-0.52)	0.11	59.94
Not specified	429	1	1	0.00	(0.00-0.19)	1.00	0.00
**Sample type[6]**							
Fecal	5639	24	47	0.30	(0.00-0.97)	<0.01	72.50
Both (Fecal+Autopsy)	80	2	5	0.09	(0.00-3.25)	0.97	0.00

**Diagnostic method (Crypto)**[7]							
ELISA	93	1	4	0.00	(0.00-1.48)	0.97	0.00
RA	1370	3	3	0.00	(0.00-2.31)	0.15	57.92
Several	2160	11	23	1.58	(0.35-3.38)	<0.01	72.96
MS	2096	11	22	0.00	(0.00-0.08)	0.01	35.34
**Diagnostic method (ETEC)**[7]							
ELISA	183	2	5	1.25	(0.00-4.20)	0.36	4.42
RA	1988	5	9	2.22	(0.04-6.29)	<0.01	87.25
Several	1426	10	21	0.87	(0.08-2.21)	<0.01	44.39
Agglutination	2122	9	17	0.00	(0.00-0.00)	0.06	33.17
**Study type**[8]							
Case-control	2637	11	22	0.28	(0.00-1.39)	<0.01	75.93
Case	3030	14	29	0.32	(0.00-1.20)	<0.01	58.80
Other	52	1	1	0.00	(0.00-2.48)	1.00	0.00

[1] Geographical region = Individual countries were assigned to Europe, Australia, West Asia, East Asia, South Asia, North America, South America and Africa.
[2] Period = Sample period described the begin and end of sampling in the studies. In case that the sampling period was not reported the publication data was used.
[3] Herd type = More than one herd types was assigned to the category "mixed" and the category "not specified" included studies, which not defined the herd type.
[4] Age class = If available in the studies, the median or mean of the animal age was used. If the median or mean of the animal age was not available, the center of the published age range was calculated and were clustered in 7-day periods.
[5] Health status = The health status cover animals with diarrhoea, normal (=without diarrhoea), mixed (=both diarrhoea and normal) and studies without information to the health status were assigned in the category "not specified".
[6] Sample type = This category included fecal samples or fecal and autopsy samples.
[7] Diagnostic method = This category covered ELISA= Antigen enzyme-linked immunosorbent assay; RA= Lateral flow immunochromatographic assay; Several= covered more than one diagnostic methods for screening and confirmation of laboratory results; MS= Acid-fast staining; Agglutination = Bacterial culture followed by slide agglutination or latex agglutination.
[8] Study type = Case-control studies covered studies which tested diarrheic and normal calves and/or studies sampled animals in several regions of a country; Case studies incorporated studies which tested animals with diarrhoea and/or and/or studies sampled animals in several regions of a country.
[9] Qep = The Q statistic and its p-value serve as a test of significance.
[10] I^2 = The ratio of true heterogeneity to total variation in calculated effects, expressed in %.

Table S5. Estimated mean prevalence (%) of the pathogens in the various countries, shown in Figure 1.

Country	Crypto	BRV	BCoV	ETEC	Crypto-BRV	Crypto-BCoV	Crypto-ETEC
Germany	48,67	39,45	19,02	4,24	16,28	4,29	0,00
Ireland	44,29	38,63	9,12	14,08	16,70	3,39	3,39
Italy	57,16	32,18	3,56	3,75	10,63	0,00	3,39
Netherlands	27,06	16,72	1,73	1,45	6,38	NA	NA
Spain	43,18	41,67	24,60	10,07	15,39	0,96	2,96
Sweden	20,89	15,09	1,84	2,71	1,17	0,76	0,00
Switzerland	42,71	36,57	3,61	4,61	15,57	1,32	0,50
Turkey	44,57	32,17	4,27	18,01	16,61	1,74	4,71
United Kingdom	23,75	36,35	14,88	2,07	6,52	4,34	0,00

Figure S1. Studies incorporated in the presented study.

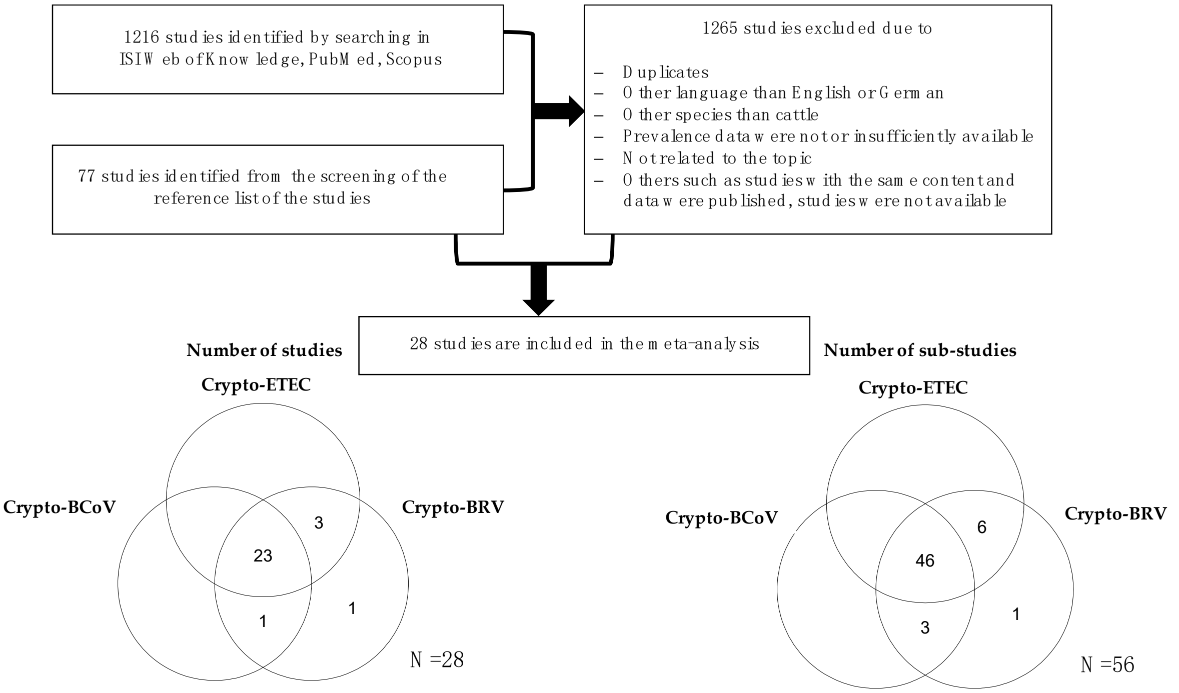

Figure S2. Forest plot of prevalence with *Cryptosporidium* spp. (Crypto) ordered by health status and publication year of the studies.

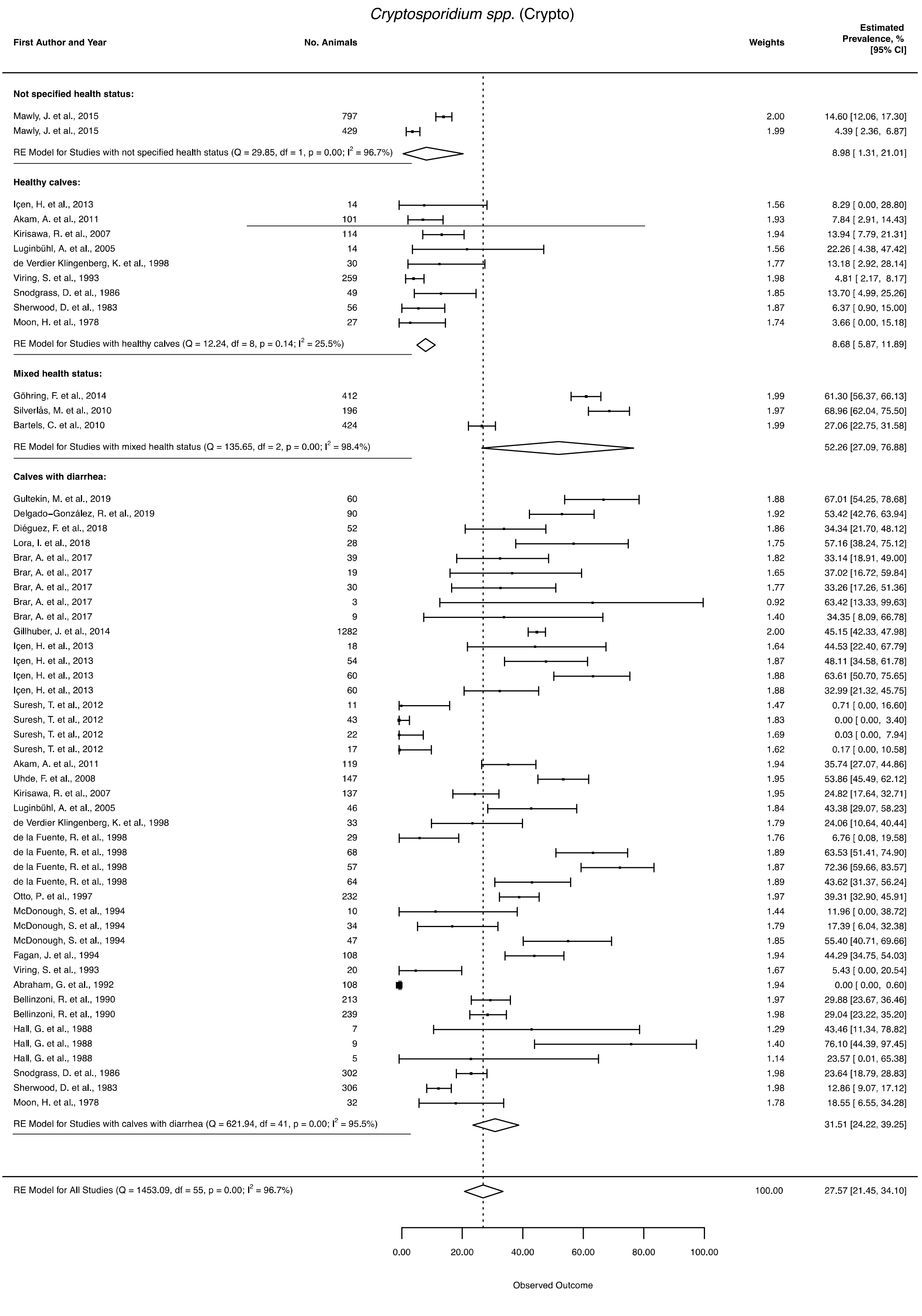

Figure S3. Forest plot of prevalence with *Cryptosporidium* spp. (Crypto) and bovine rotavirus (BRV) ordered by health status and publication year of the studies.

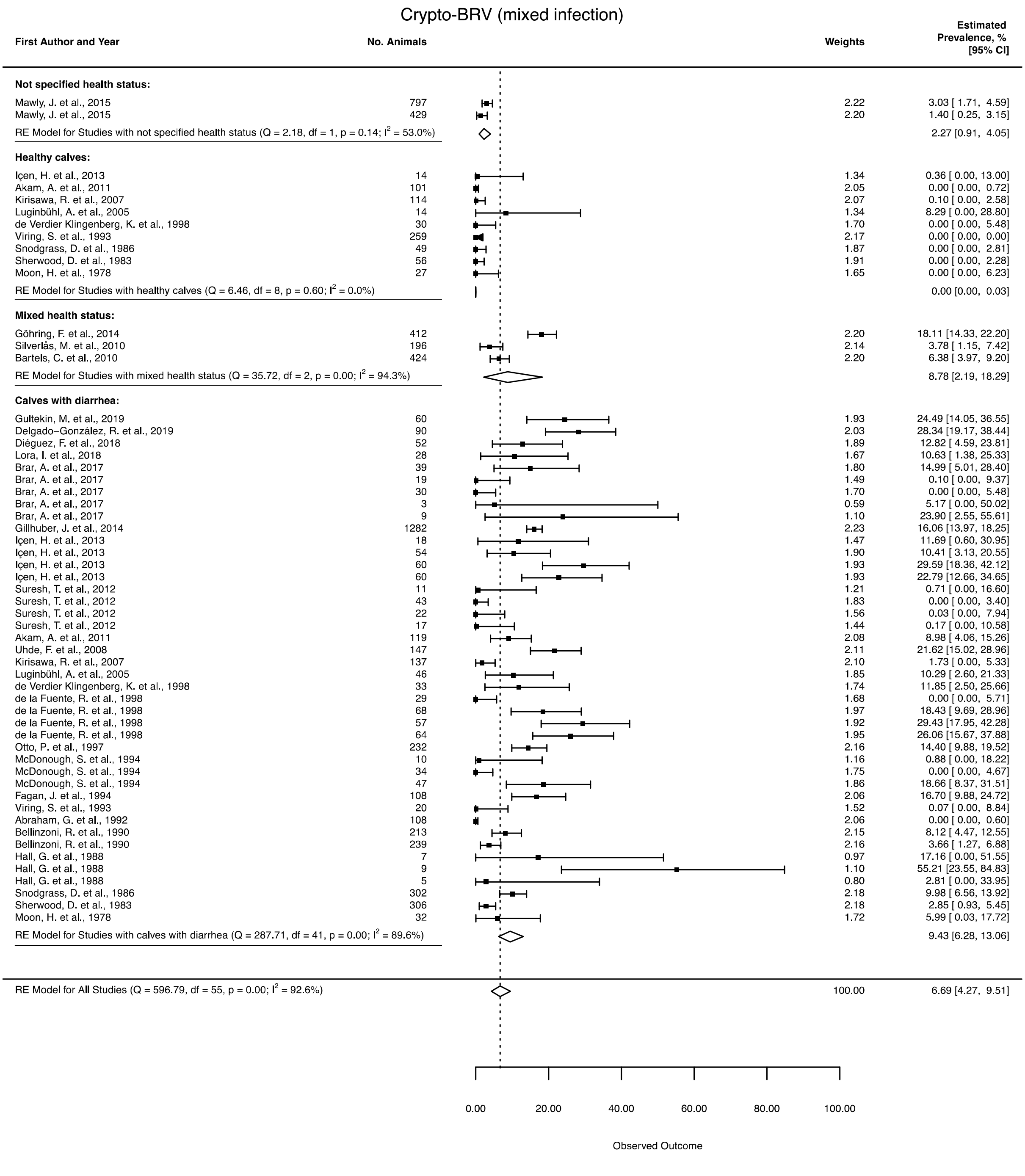

Figure S4. Forest plot of prevalence with *Cryptosporidium* spp. (Crypto) and bovine coronavirus (BCoV) ordered by health status and publication year of the studies.

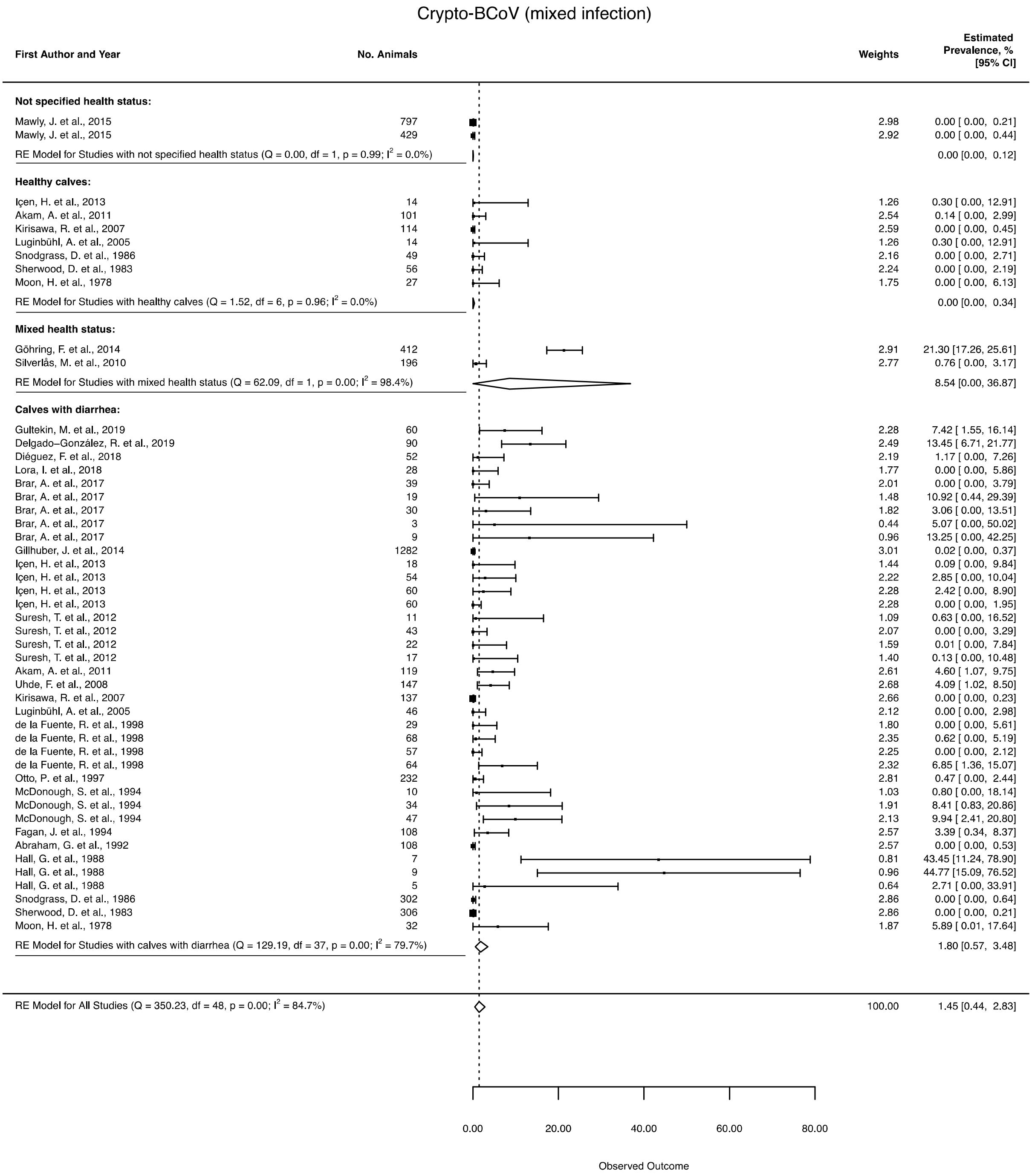

Figure S5. Forest plot of prevalence with *Cryptosporidium* spp. (Crypto) and *Escherichia coli* F5 (K99) (ETEC) ordered by health status and publication year of the studies.

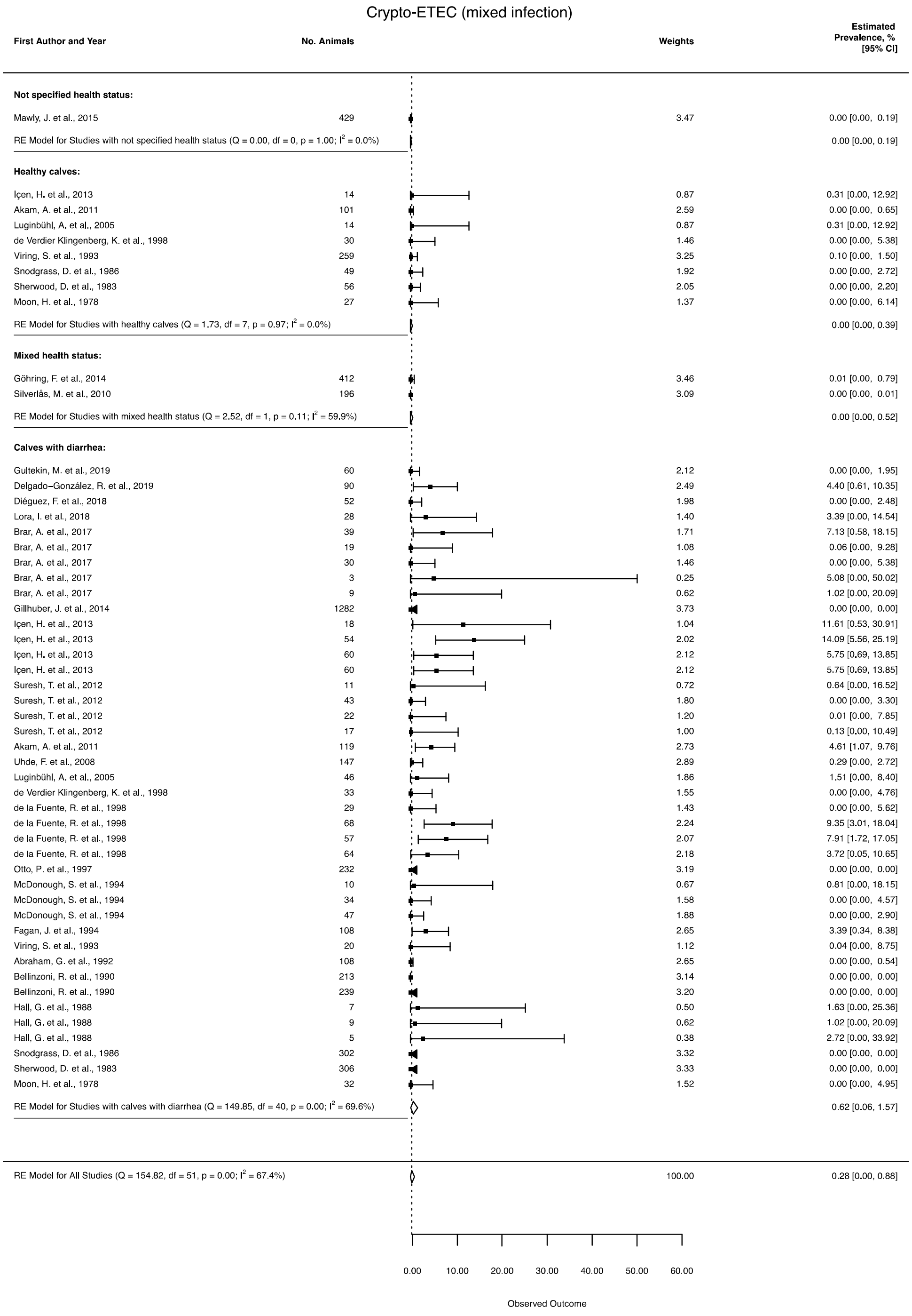

Figure S6. Case influence diagnostic analysis (a) and funnel plot (b) of *Cryptosporidium* spp. (Crypto) and the identified outliers (shown as red circles). N.B. In case of Crypto-BRV no outliers were identified. The regression test for funnel plot asymmetry in the meta-analysis indicated no publication bias for Crypto (z = -0.33; p = 0.73).

a)

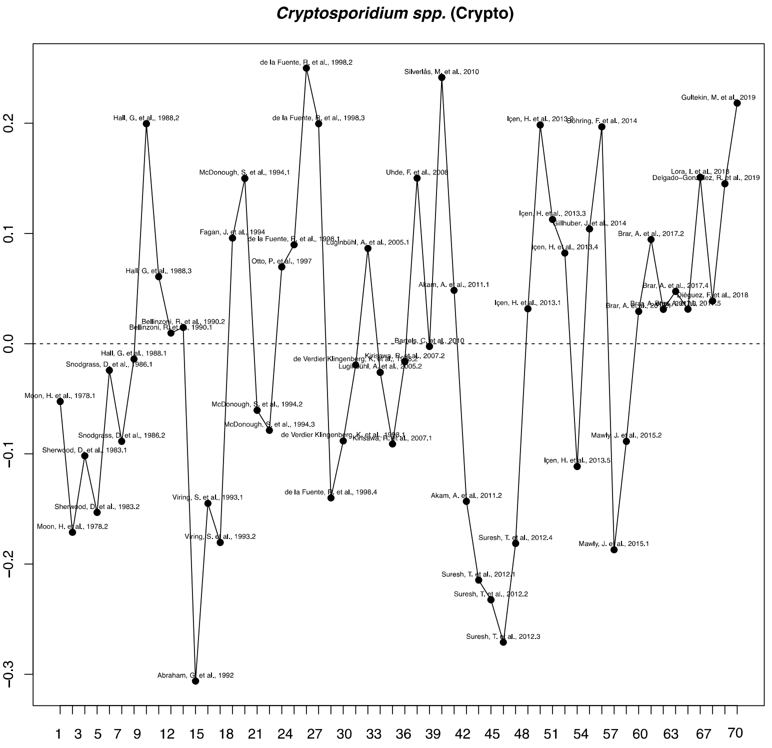

b)

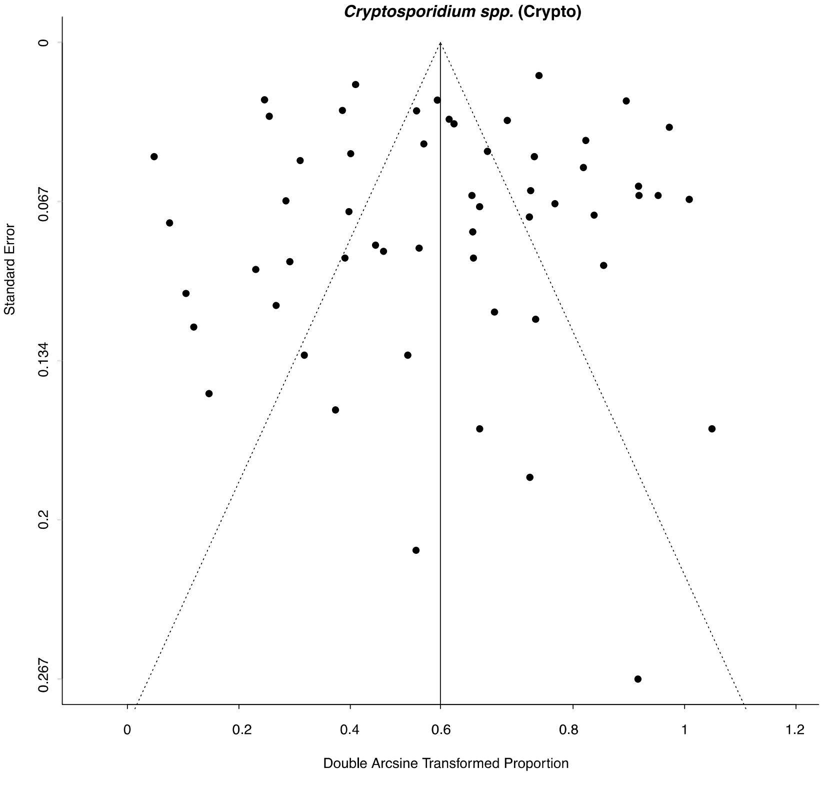

Figure S7. Case influence diagnostic analysis (a) and funnel plot (b) of *Cryptosporidium* spp. (Crypto) and bovine rotavirus (BRV) and the identified outliers (shown as red circles). N.B. In case of Crypto-BRV no outliers were identified. The regression test for funnel plot asymmetry in the meta-analysis indicated no publication bias for Crypto-BRV (z = -0.25; p = 0.79).

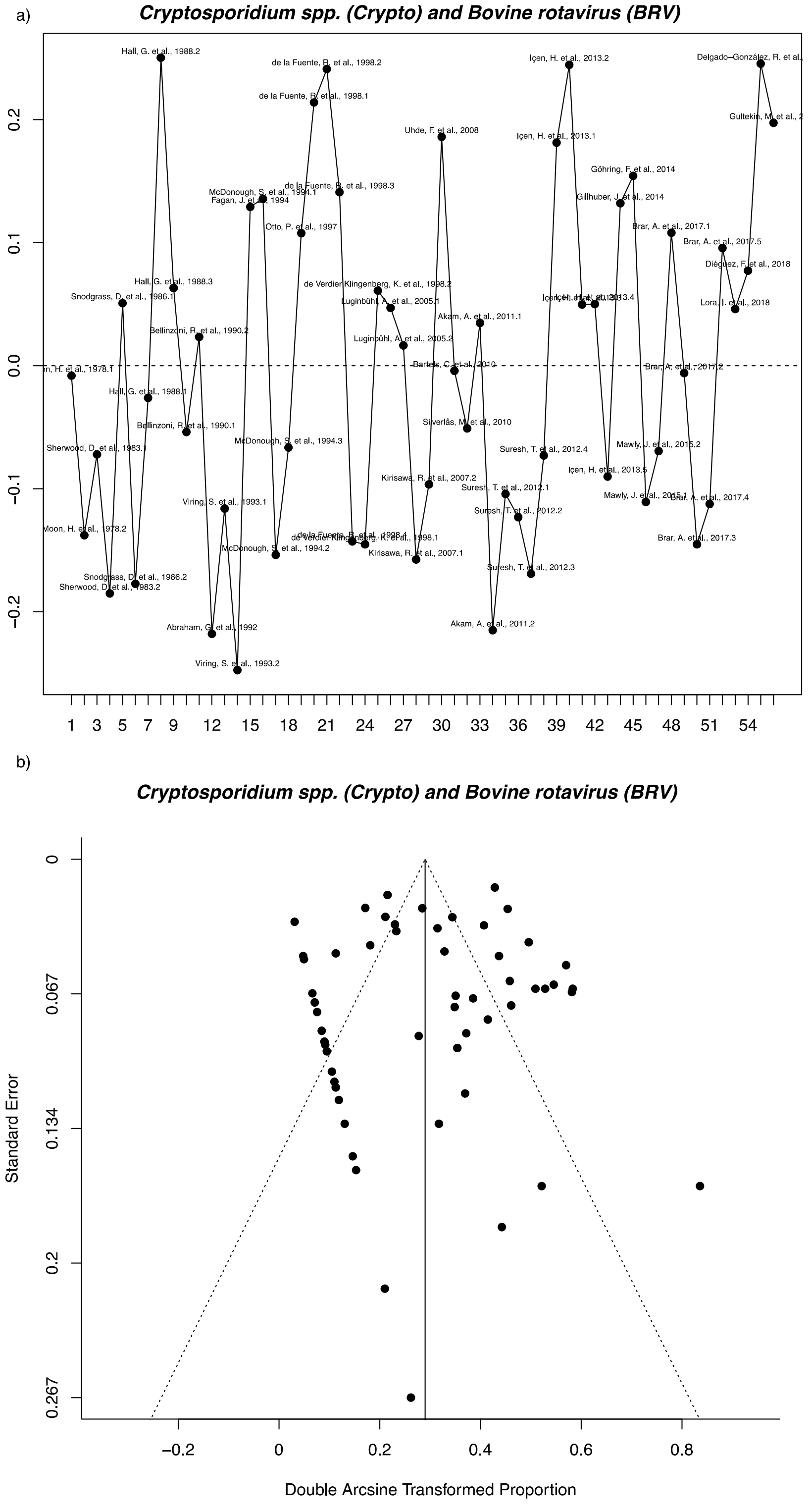

Figure S8. Case influence diagnostic analysis (a) and funnel plot (b) of *Cryptosporidium spp.* (Crypto) and bovine coronavirus (BCoV) and the identified outliers (shown as red circles). The regression test for funnel plot asymmetry in the meta-analysis indicated no publication bias for Crypto-BCoV (z = 1.89; p = 0.05).

a)

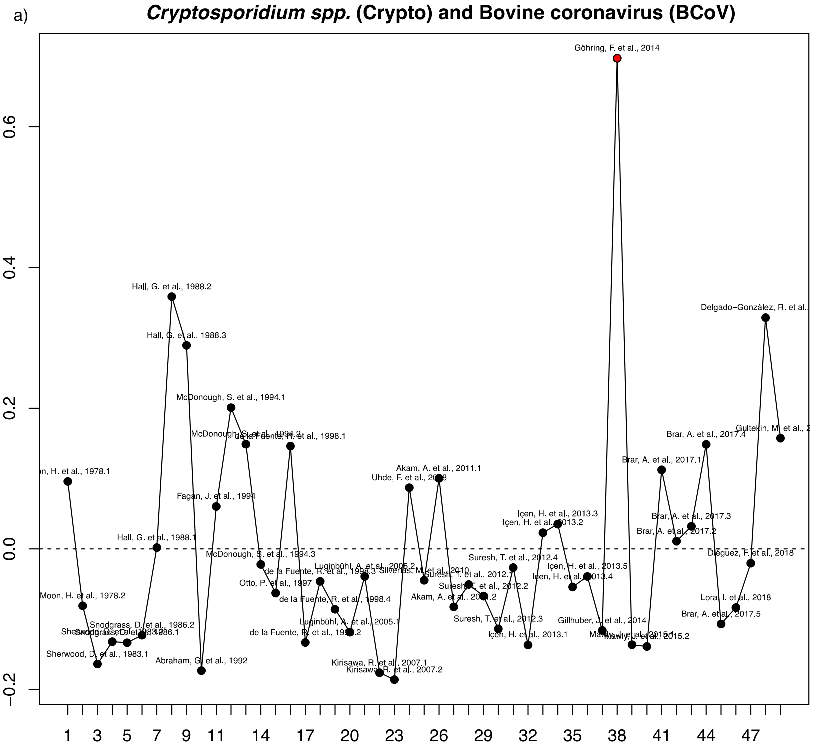

b)

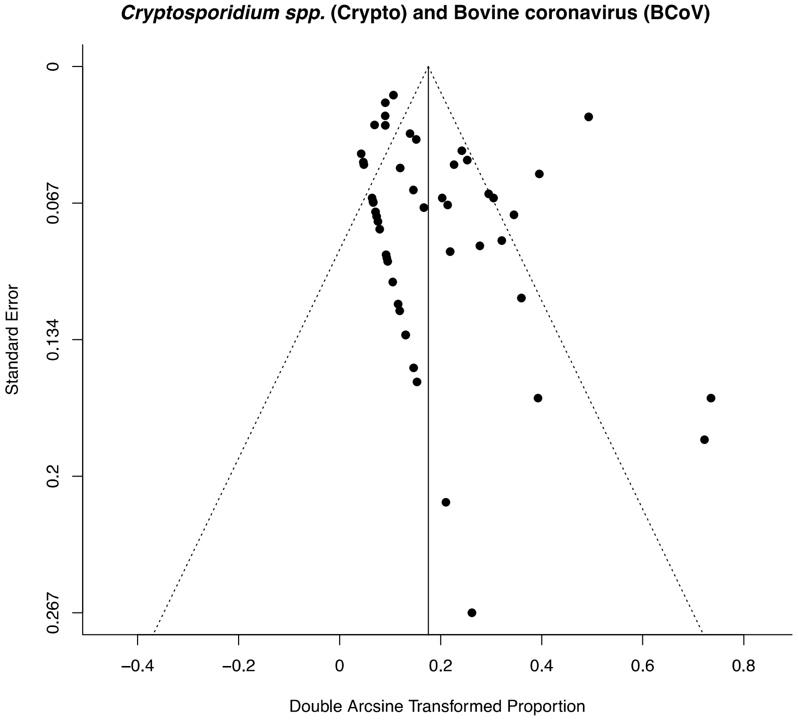

Figure S9. Case influence diagnostic analysis (a) and funnel plot (b) of *Cryptosporidium spp.* (Crypto) and *Escherichia coli* F5 (K99) (ETEC) and the identified outliers (shown as red circles). The regression test for funnel plot asymmetry in the meta-analysis indicated a publication bias for Crypto-ETEC (z =2.30; *p* =0.02).

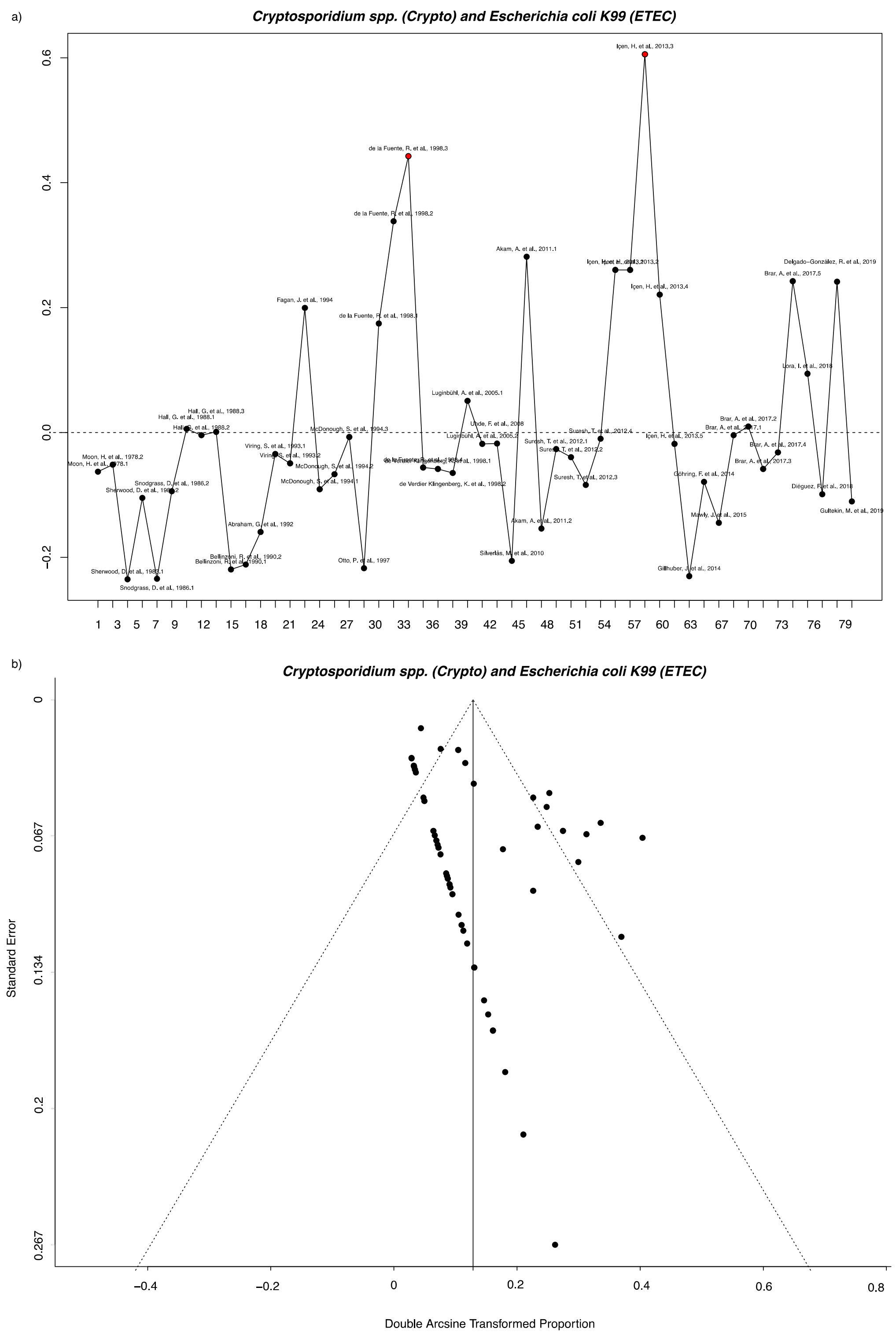

Supplement: Supplementary file 1 [file animals-11-01786-s001.zip › animals-1227098-supplementary.pdf]
